# Protective Immunity Induced by TgMIC5 and TgMIC16 DNA Vaccines Against Toxoplasmosis

**DOI:** 10.3389/fcimb.2021.686004

**Published:** 2021-09-14

**Authors:** Yu-Chao Zhu, Li-Juan Ma, Ji-Li Zhang, Jian-Fa Liu, Yong He, Ji-Ye Feng, Jia Chen

**Affiliations:** ^1^Department of Radiology, The Affiliated Hospital of Medical School of Ningbo University, Ningbo University School of Medicine, Ningbo, China; ^2^Department of Integrated Chinese and Western Medicine Oncology, The Affiliated People’s Hospital of Ningbo University, Ningbo, China; ^3^Immunology Innovation Team, Ningbo University School of Medicine, Ningbo, China; ^4^Department of Hepatobiliary Surgery, The Affiliated People’s Hospital of Ningbo University, Ningbo, China

**Keywords:** *Toxoplasma gondii*, DNA vaccine, MIC5, MIC16, parasite

## Abstract

*Toxoplasma gondii* is an obligate intracellular parasite, which is responsible for a widely distributed zoonosis. Effective vaccines against toxoplasmosis are necessary to protect the public health. The aim of this study is to evaluate the immune efficacy of DNA vaccines encoding TgMIC5 and TgMIC16 genes against *T. gondii* infection. The recombinant plasmid pVAX-MIC5 and pVAX-MIC16 were constructed and injected intramuscularly in mice. The specific immune responses and protection against challenge with *T. gondii* RH tachyzoites were evaluated by measuring the cytokine levels, serum antibody concentrations, lymphocyte proliferation, lymphocyte populations, and the survival time. The protection against challenge with the *T. gondii* RH tchyzoites and PRU cysts was examined by evaluation of the reduction in the brain cyst burden. The results indicated that immunized mice showed significantly increased levels of IgG, IFN-γ, IL-2, IL-12p70, and IL-12p40 and percentages of CD4^+^ and CD8^+^ T cells. Additionally, vaccination prolonged the mouse survival time and reduced brain cysts compared with controls. Mouse groups immunized with a two-gene cocktail of pVAX-MIC5 + pVAX-MIC16 were more protected than mouse groups immunized with a single gene of pVAX-MIC5 or pVAX-MIC16. These results demonstrate that TgMIC5 and TgMIC16 induce effective immunity against toxoplasmosis and may serve as a good vaccine candidate against *T. gondii* infection.

## Introduction

*Toxoplasma gondii*, an intracellular protozoan parasite, is able to infect almost all warm-blooded animals and humans (Mohamed-Ali et al., 2017; Florence and Marie-Laure, 2012). *T. gondii* is one of the most widespread parasites, with one-third of the world’s population estimated to be chronically infected (Dubey, 2009; Pappas et al., 2009). In common, intermediate hosts including humans acquire infection by eating raw meat, and exposure to soil containing oocysts excreted by the only final host of *T. gondii*, cats. Toxoplasmosis is usually characterized as asymptomatic or subclinical infection in immunocompetent individuals, but it can cause serious complications or even a fatal disease in immunocompromised populations (e.g., HIV infected patients, cancer patients, and transplant recipients) (Saki et al., 2013; Wang et al., 2017; Elsheikha et al., 2020). If pregnant women acquire primary infection with *T. gondii*, fetal abortion may occur (Elsheikha, 2008; Flegr et al., 2014; Elsheikha et al., 2020). Also, *T. gondii* infection can lead to abortion and stillbirth in livestock, including goats and sheep, thereafter causing financial losses to the livestock industry (Tenter et al., 2000). Unfortunately, currently available drugs can only target the tachyzoite stage of *T. gondii*, while no effective chemotherapy can eliminate tissue cysts that persist life-long in the infected hosts (Wang et al., 2019). Therefore, immunoprophylaxis is considered to be an alternative approach for the prevention and control of toxoplasmosis (Zhang et al., 2013; Zhang et al., 2015).

The licensed vaccine which has been used for ovine toxoplasmosis (Toxovax) is obtained from the non-cyst forming S48 strain. However, it is unknown whether the vaccination has any effect on the tissue cyst formation and thus it is inapplicable to humans due to safety concerns (Erik et al., 2009; Burrells et al., 2015). Until now, no available vaccine has been used for protection against *T. gondii* infection in other animal species or humans (Wang et al., 2019). Hence, safe and effective vaccines would be extremely valuable for controlling of *T. gondii* infections in animals and humans. There is an urgent need to find a novel, effective, and safe vaccine to combat *T. gondii* infections.

The DNA vaccine is well known as safe, convenient, low cost, and can induce vigorous cellular and humoral immune responses (Margaret, 2011). A number of *T. gondii* excretory secretory antigens have been tested as DNA vaccine candidates (e.g. TgGRA24, TgGRA25, TgROP5, TgROP18, and TgDOC2C, etc.) and they showed effective immunity against *T. gondii* infection, with inductive Th1 type and CD8+ cytotoxic T-lymphocyte responses, and prolonged survival time in mice after infection with *T. gondii* (Zhang et al., 2018; Xu et al., 2019; Zhu et al., 2020)., Some microneme proteins (MICs) have also been shown to have immunogenic capabilities (Dodangeh et al., 2019). TgMIC5 is one of a large member of microneme proteins, which can regulate the activity of proteases and it is related to the proteolytic susceptibility of other microneme protein substrates (Susannah et al., 2006). TgMIC16 is indispensable for binding to aldolase and is also involved in rhomboid cleavage and trafficking signals during *T. gondii* invasion (Lilach et al., 2010). These important features suggest that TgMIC5 and TgMIC16 proteins may be good DNA vaccine candidates, which can elicit protective immunity against infection with *T. gondii*.

The objectives of this study were to assess the potential of TgMIC5 and TgMIC16 as DNA vaccine candidates against infection with *T. gondii*. We constructed the eukaryotic plasmids, pVAX-MIC5 and pVAX-MIC16, and determined the protective immunity elicited by the DNA vaccine based on pVAX-MIC5 and/or pVAX-MIC16 against acute and chronic *T. gondii* infection in Kunming mice.

## Materials and Methods

### Mice and Parasite

Seven-week-old specific-pathogen-free (SPF) Kunming female mice were purchased from Zhejiang Laboratory Animal Center, Hangzhou, China. All mice in this study were handled in strict accordance with the Animal Ethics Procedures and Guidelines of the People’s Republic of China. This study was approved by the Animal Ethics Committee of Ningbo University (permission: SYXK(ZHE)2013-0190).

*T. gondii* tachyzoites of the RH strain (Type I) and cysts of the PRU strain (Type II) were used in this study, which were propagated and harvested according to the methods described in previous studies (Chen et al., 2016). The obtained tachyzoites were used for the preparation of *Toxoplasma* lysate antigen (TLA) and also for total RNA extraction (RNAprep Pure Tissue Kit, TIANGEN, China), as described previously (Chen et al., 2015). In brief, prior to centrifugation, the cellular lysate was prepared by disruption of *T. gondii* RH tachyzoites using three cycles of freezing at −20°C and thawing, followed by sonication on ice at 60 W/s. Then, TLA was obtained from the supernatants, and then pooled and sterile-filtered with 0.2 μm nitrocellulose filters (Sartorius, Germany).

### Construction of the Eukaryotic Expression Plasmid

The coding sequences of the TgMIC5 and TgMIC16 gene were amplified by polymerase chain reaction (PCR) from tachyzoite cDNA of *T. gondii* RH strain, using two pairs of oligonucleotide primers: (MIC5F, forward primer: 5′-GGGGTACCATGCTGCGACCTACTGTT -3′); (MIC5R, reverse primer: 5′-GCCTAGACTATGCGAGTTTCACCTC -3′); (MIC16F, forward primer: 5′-GGGGTACCATGCTGCGACCTACTGTT -3′); (MIC16R, reverse primer: 5′-GCTCTAGACTATGCGAGTTTCACCTC -3′). The two obtained PCR products were inserted into pMD18-T vector (TaKaRa, China) and sequenced in both directions to ensure fidelity and the construction of pMD-MIC5 and pMD-MIC16. The TgMIC5 and TgMIC16 fragments were cleaved by *Xba* I and *Kpn* I (TaKaRa, China) from pMD-MIC5 and pMD-MIC16 and then subcloned into the pVAX I vector. The recombinant plasmids pVAX-MIC5 and pVAX-MIC16 were transferred into *E. coli* DH5α, and positive clones were selected *via* double restriction enzyme digestion and DNA sequencing. Then, these two plasmids were purified by anion exchange chromatography (EndoFree plasmid giga kit, Qiagen Sciences, Maryland) following the manufacturer’s instructions, and were diluted with sterile phosphate buffered saline (PBS) and stored at –20°C until use. The concentration of pVAX-MIC5 and pVAX-MIC16 was determined with spectrophotometer at OD_260_ and OD_280_.

### Expression of pVAX-MIC5 and pVAX-MIC16 Plasmid *In Vitro*

Indirect immunofluorescence assay (IFA) was used to detect the expression of pVAX-MIC5 and pVAX-MIC16 *in vitro*, followed by pVAX-MIC5 and pVAX-MIC16 transfection into 293-T cells using Lipofectamine 2000 reagent (Invitrogen, Carlsbad, CA) according to the manufacturer’s instructions as previously described (Chen et al., 2015). In brief, 48 h post-transfection with pVAX-MIC5 or pVAX-MIC16, cells were fixed with 100% chilled acetone for 30 min. Following washing with PBS-0.1% Triton-X-100 (PBST), anti-*T. gondii* polyclonal antiserum (1:50 dilution in PBS-0.1% Triton-X-100 [PBST]) and fluorescein isothiocyanate (FITC)-labeled donkey-anti-goat IgG (Proteintech Group Inc., Chicago) were added to each well. Finally, the stained monolayers were covered with glycerin and then fluorescence was imaged using a Zeiss Axioplan fluorescence microscope (Carl Zeiss, Germany). 293-T cells transfection with empty pVAX I was used as the negative control.

### Immunization and *T. gondii* Challenge in Mice

The experiment groups (25 mice/group) consisting of mice immunized with 100 μL (1 μg/μL) plasmids diluted in PBS, including pVAX-MIC5, pVAX-MIC16, and pVAX-MIC5 + pVAX-MIC16, respectively, which were injected intramuscularly (i.m.) three times at 2-week intervals. Control groups include mice that received 100 μL PBS (negative control group), 100 μg empty pVAX I plasmid (vector control group; 1 μg/μL), and no treatment (blank control). Blood from each group of mice was collected from the tail vein at 0, 2, 4, and 6 weeks prior to immunization. Sera were collected after centrifugation for 5 min at 4000 g and stored at -20°C until further study.

The mice (10 per group) in all groups were intraperitoneally (i.p.) inoculated with 1×10^3^ tachyzoites of RH strain 2 weeks after the third immunization, and the survival was recorded daily until 35 days. The mice (another 6 mice per group) were inoculated orally with 20 cysts of the PRU strain at 2 weeks after the third immunization, the mouse brains were removed and homogenized in 1 ml of PBS, and the mean number of brain cysts was determined under a microscope (40 × objective) using three aliquots of 20 μL of brain homogenates. All samples were analyzed in triplicate. Two weeks after the last immunization, a total of 9 mice per group were sacrificed and their splenocytes were used for cytokine measurements (3 mice), lymphocyte proliferation assay (3 mice), and flow cytometric analysis (another 3 mice).

### Measurement of Antibody Responses

According to the manufacturer’s instruction of SBA Clonotyping System-HRP Kit (Southern Biotech Co., Ltd, Birmingham, United Kingdom) and as described in methods previously (Chen et al., 2015), ELISA was used to determine the levels of IgG, IgG1, and IgG2a in sera samples collected at 0, 2, 4, and 6 weeks. In brief, the microtiter plates were coated with 100 μL (10 μg/mL) TLA diluted in PBS and incubated at 4°C overnight. The plates were then washed three times by PBS containing 0.05% Tween20 (PBST) and blocked by PBS containing 1% BSA for 1 h at 37°C. After washing with PBS, the plates were incubated with the sera diluted by PBS for 1 h at room temperature. The anti-mouse-IgG, IgG1, and IgG2a horseradish peroxidase (HRP)-conjugated antibodies were added to each well for 1 h at 37°C. The efficient binding was visualized by incubation with substrate solution (100 μL; pH 4.0; 1.5% ABTS, 1.05% citrate substrate buffer, 0.03% H_2_O_2_) for 30 min at room temperature. The absorbance was measured at 405 nm using the ELISA reader (Bio-TekEL×800, U.S.). All samples were analyzed in triplicate.

### Lymphoproliferation Assay

Splenocyte suspensions from 3 mice in each group were prepared by pushing the spleens through a wire mesh, and purified through removing the red blood cells by using RBC erythrocyte lysis buffer, and then resuspended in DMEM medium supplemented with 10% fetal bovine serum (FBS; Gibco, Carlsbad, CA). In brief, 3×10^6^ cells per well were cultured in 96-well Costar plates stimulated with TLA (10 μg/ml), concanavalin A (ConA; positive control; 5 μg/ml; Sigma, St. Louis, MO), or medium alone (negative control), at 37°C for 72 h with 5% CO_2._ Then, 10 ul of 3-(4,5-dimethylthylthiazol-2-yl)-2,5-diphenyltetrazolium bromide (MTT, 5 mg/ml, Sigma, St. Louis, MO) was added to each well and incubated for 4 h. The stimulation index (SI) was calculated as per the following formula: stimulation index (SI) = (OD570 TLA/OD570 Control):(OD570 ConA/OD570 Control). All samples were analyzed in triplicate, which were obtained from 3 different mice.

### Flow Cytometry Analysis

The percentages of CD4+ and CD8+ T lymphocytes in the purified splenocytes obtained from the mice after the last immunization were analyzed by flow cytometry. Splenocytes suspensions were prepared as mentioned previously and then stained with fluorochrome-labeled mAbs including PE-CD3, APC-CD4, and FITC-CD8 (eBioscience, United States) at 4°C for 30 min in the dark. After washing with 2 ml PBS, the cultures were fixed with FACScan buffer (PBS containing 1% FBS and 0.1% sodium azide) and 2% paraformaldehyde. The samples were run in a FACScan flow cytometer (BD Bio-sciences, United States) and then were analyzed for fluorescence by SYSTEM II software (Coulter). All samples were run in triplicate independently, which were obtained from three different mice.

### Cytokine Assays

Spleen cells grown in 96-well plates were restimulated with TLA (10 μg/ml) or medium alone (negative control). The commercial ELISA kits (Biolegend, United States) were used to detect the levels of cytokines harvested from cell-free supernatants, which were assayed for IL-2, IL-4, and IL-12p40 levels at 24 h, for IL-10 levels at 72 h, and for gamma interferon (IFN-γ) and IL-12p70 levels.

### Statistical Analysis

All statistical analyses were conducted with SPSS17.0 Data Editor (SPSS, Inc., Chicago, IL). The differences (e.g., antibody and percentage of CD4^+^ and CD8^+^ T cells) between all the groups were compared by one-way ANOVA. The level of significant difference between groups was considered significantly different if *P<*0.05. The survival time after challenge with RH strain was conducted with Kaplan-Meier.

## Results

### Expression of pVAX-MIC5 and pVAX-MIC16 Plasmid *In Vitro*

**Figure 1:**
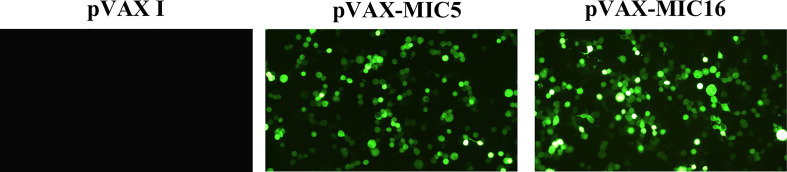
Detection of the recombinant TgMIC5 and TgMIC16 protein expressed in 293T cells. 293T cells were transfected with empty pVAX I, pVAX-MIC5, or pVAX-MIC16.

Specific green fluorescence was observed in the 293T cells transfected with pVAX-MIC5 and pVAX-MIC16, while no fluorescence was observed in cells transfected with the empty pVAX I (**Figure 1**).

### Antibody Response and Isotype Determination

**Figure 2:**
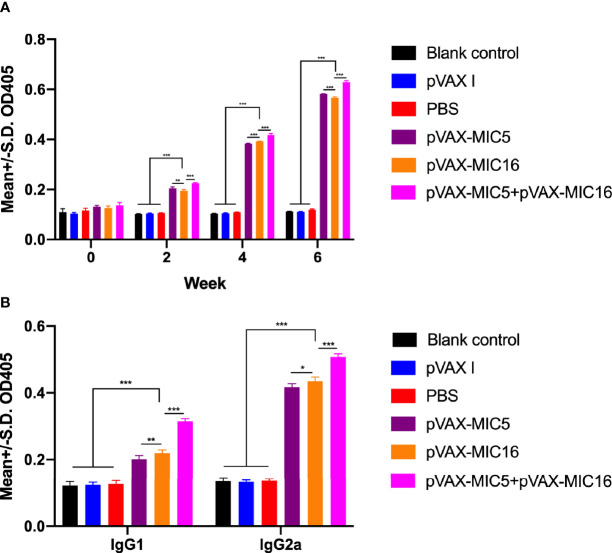
Detection of specific humoral immune responses induced by DNA immunization with single or multiple genes. **(A)** Determination of IgG antibodies in the sera of Kunming mice at 0, 2, 4, and 6 weeks; **(B)** detection of IgG1 and IgG2a antibodies in immunized mice 2 weeks after the last immunization. *P < 0.05, **P < 0.01, ***P < 0.001. Data are presented as the means ± SD.

The levels of total IgG and subclasses IgG1 and IgG2a were detected by ELISA. As shown in **Figure 2A**, a higher IgG titer was detected in the serum of mice vaccinated with single or two genes than that in three control groups. Also, pVAX-MIC5 + pVAX-MIC16 increased the antibody levels induced by DNA immunization than with pVAX-MIC5 or pVAX-MIC16. As shown in **Figure 2B**, the ratios of IgG2a/IgG1 in all immunized groups were higher, especially in pVAX-MIC5 + pVAX-MIC16 group compared with the control groups (*p* < 0.05).

### Evaluation of the Proliferative Response in Spleen Cells

**Figure 3:**
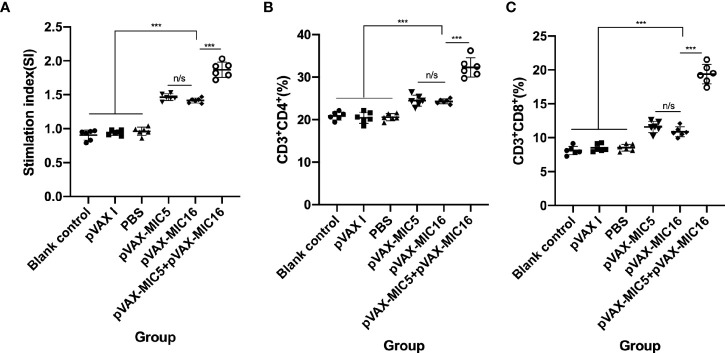
Splenocyte proliferative response and the percentages of CD4^+^ and CD8^+^ of T cells in immunized and control mice two weeks after the challenge. **(A)** Lymphocyte proliferation stimulation index (SI). **(B, C)** Determination of the percentages of CD4^+^ or CD8^+^ T cells in immunized and control mice. ***P < 0.001,“n/s”, no significant. Data are presented as the means ± SD.

The proliferative activity in splenocytes was assayed by MTT. As shown in **Figure 3A**, the SI in mice vaccinated with pVAX-MIC5 + pVAX-MIC16 was higher than that in mice immunized with a single-gene plasmid or the control groups (*P*<0.05). In contrast, mice immunized with pVAX-MIC5 or pVAX-MIC16 induced a higher SI than the control groups. However, no significant difference was observed in the control groups (*P* > 0.05).

### The Percentages of CD4^+^ and CD8^+^ T Lymphocytes

Similar to the results mentioned previously, the percentages of CD4^+^ T cells were significantly increased (*P*<0.05) in the immunized mice compared to that in the control groups. The mice immunized with pVAX-MIC5 + pVAX-MIC16 induced higher percentages of CD4^+^ T cells than that in mice immunized with a single-gene plasmid (pVAX-MIC5 or pVAX-MIC16) (**Figure 3B**). However, there was no significant difference among the three control groups (*P*>0.05). Similar to the results of the percentages of CD4^+^ T cells, the percentages of CD8^+^ T cells showed obvious increase in all immunized mice in comparison with that in control groups (**Figure 3C**) (*P* < 0.05).

### Cytokine Production

**Figure 4:**
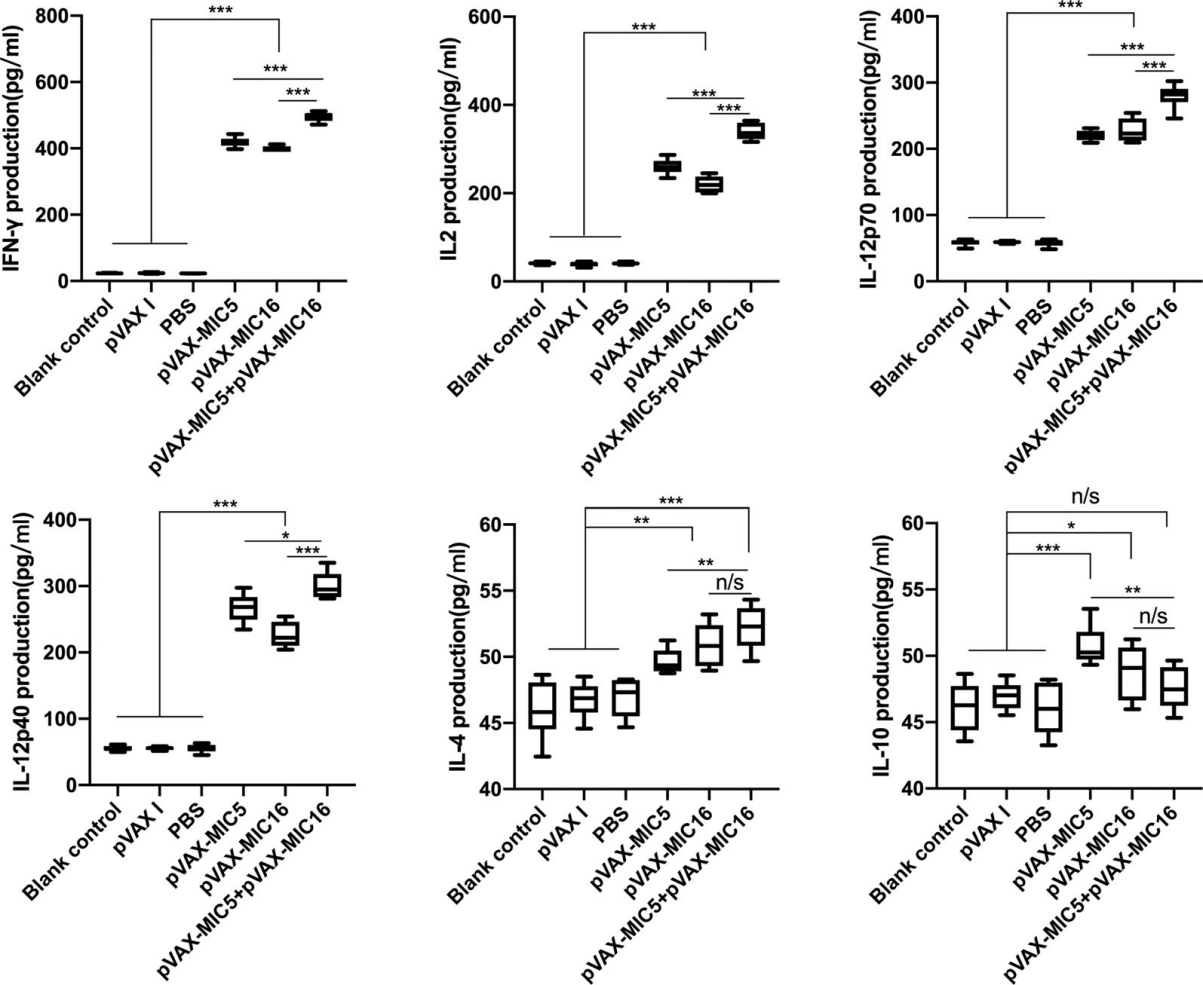
Cytokine production by splenocytes of mice immunized with single or multiple genes. *P < 0.05, **P < 0.01, ***P < 0.001. “n/s”, no significant. Data are presented as the means ± SD.

Splenocyte supernatants were obtained at 2 weeks after the last immunization, which were used to assess the levels of IFN-γ, IL-2, IL-12p70, IL-12p40, IL-4, and IL-10 in mice. As shown in **Figure 4**, the higher levels of IL-12p70 and IL-12p40, especially IFN-γ and IL-2 in DNA immunized mice, were detected in comparison to that in the controls (*P*<0.05). The mice immunized with pVAX-MIC5 + pVAX-MIC16 showed higher levels of these cytokines than the groups of mice immunized with a single-gene plasmid (pVAX-MIC5 or pVAX-MIC16). Regarding the production of IL-4 and IL-10, a slight increased production of IL-4 and IL-10 was observed in all the immunized groups compared with that in controls (*P*>0.05).

### Protective Efficacy of DNA Vaccination Against Acute and Chronic *T. gondii* Infection

**Figure 5:**
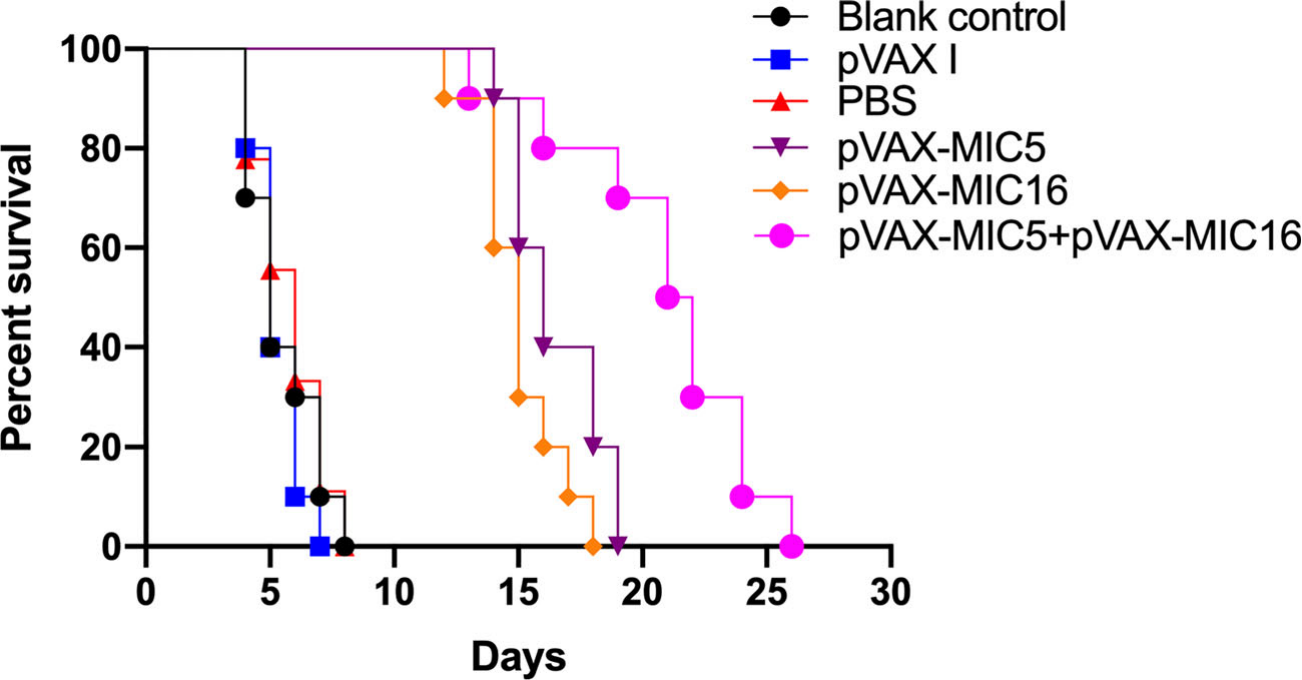
The survival rate of immunized Kunming mice challenged with 1 × 10^3^ tachyzoites 2 weeks after the last immunization.

**Figure 6:**
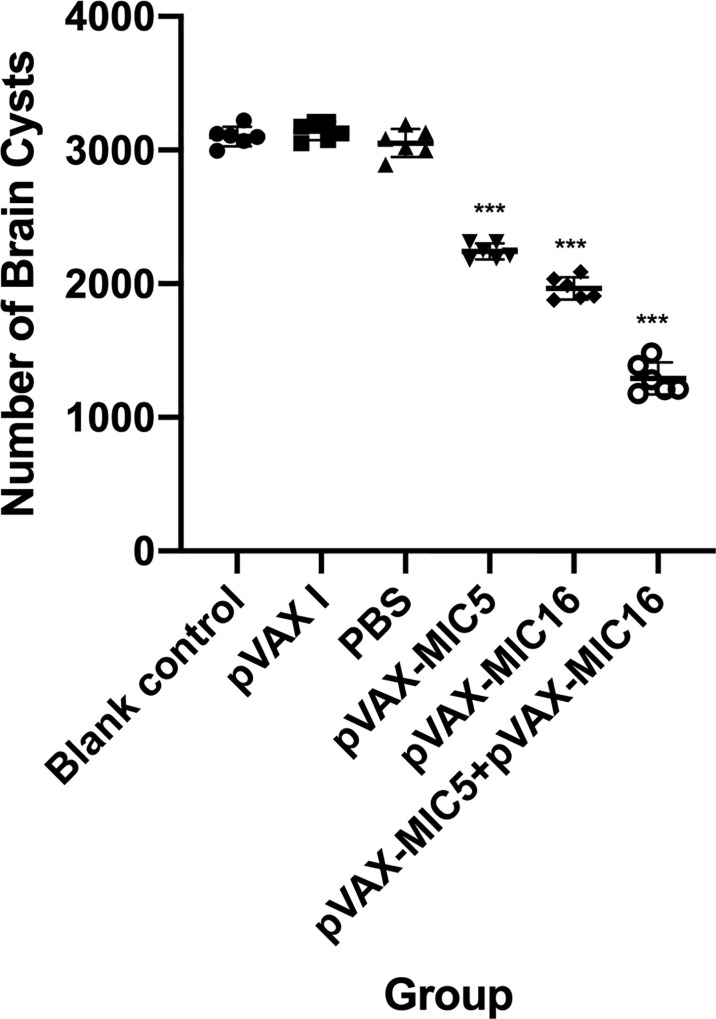
Protection against chronic toxoplasmosis in immunized mice 2 weeks after the final booster immunization. The bars represent the mean cyst burden per mouse brain after oral challenge with 20 cysts of the PRU strain. Cyst load was determined from whole brain homogenates of mice 4 weeks after challenge. Data are means ± SD (representative of three experiments). ***p < 0.001, compared with the control groups.

To evaluate the protective efficacy induced by the DNA immunization, the survival curve in immunized mice after challenge of *T. gondii* RH strain as shown in **Figure 5** and this suggested that the three immunized groups had significantly prolonged survival time compared with the control groups, which died within 6 days post challenge (*P*<0.05). As shown in **Figure 6**, a significant reduction of brain cyst was observed in mice immunized with pVAX-MIC5 (28%), pVAX-MIC16 (39.44%), and especially pVAX-MIC5 + pVAX-MIC16 (48.06%) compared to the controls (*P*<0.05). No statistical difference was observed between the three control groups (*P*>0.05).

## Discussion

In recent years, DNA vaccines can evoke durable immune responses, which is critical to provide protective immunity against infection with *T. gondii* (Li and Zhou,, 2018). Previous studies have evaluated the immunoprotective efficacy of *T. gondii* MIC antigens, including TgMIC2, TgMIC2, TgMIC11, and TgMIC14 (Foroutan et al., 2018). In the present study, DNA immunization with pVAX-MIC5 and/pVAX-MIC16 has activated humoral and Th1-type cellular immune responses, which may contribute to the increased survival of mice challenged with *T. gondii* RH strain and the reduction in the brain cysts post challenge with *T. gondii* PRU strain. Additionally, these two antigen-based cocktail DNA vaccines mounted more protective immunity than a single antigen-based DNA immunization, which is consistent with studies based on vaccination with multiple antigenic peptides (Sun et al., 2014; Zhou and Wang, 2017). These results suggest that DNA immunization with TgMIC5 and TgMIC6 can induce considerable protection against acute and chronic *T. gondii* infection, demonstrating that a cocktail DNA vaccine can trigger a more potent protective immunity than that induced by single antigen-based DNA vaccine (Foroutan et al., 2018).

Following natural infection with *T. gondii*, B cells are activated and specific antibodies are produced (Sikorski et al., 2021). Therefore, efficient humoral responses are of great significance in limiting the spread of *T. gondii* tachyzoites (Sayles et al., 2000). In the present study, the significantly increased levels of anti-*T. gondii* IgG in the mice immunized with the cocktail vaccine may have contributed to limiting the infection with *T. gondii* tachyzoites, which was in line with the results from DNA immunization with TgROP18, TgROP5, or TgGRA25, TgGRA25, and TgMIC5 (Chen et al., 2015; Xu et al., 2019). Furthermore, DNA immunization with single gene vaccine, especially two-gene cocktail vaccine, significantly increased the levels of IgG2a and IgG1 compared with that in the control groups. Meanwhile, the higher ratio of IgG2a/IgG1 in immunized groups suggests that Th1-type immune responses were predominant, in accordance with previous DNA vaccination studies (Gigley et al., 2009; Matowicka et al., 2009).

T cell-mediated adaptive immune responses are critical to efficient control of *T. gondii* infection (Silva et al., 2009). In this study, a significant *T. gondii* specific splenocyte proliferation was induced in all immunized mice, suggesting an activated adaptive immune response against *T. gondii* infection, which may contribute to effective cellular immune responses against *T. gondii* infection. Generally, T helper type 1 (Th1) cells are major effectors against intracellular pathogens, including *T. gondii*, mediated by the production of Th1 type cytokines, IFN-γ, IL-12, and IL-2 (LaRosa et al., 2008; Christopher et al., 2012). IFN-γ is more critical for protective immunity than cytotoxicity-based effector functions during *T. gondii* infection (Silva et al., 2009). IL-2 is also indispensable for the development of immunological memory in T cells, depending on the expansion of the number and function in antigen-selected T cell clones (Matowicka et al., 2009). Furthermore, IL-12p40 can facilitate the proliferation of T cells and the production of IFN-γ (Matowicka et al., 2009). Additionally, IL-12p70 plays a key role in the Th1 cells immune response, and also can effectively promote the generation of Th1-biased cytokine, such as IFN-γ (LaRosa et al., 2008). The concomitant IL-10 and IL-4 responses, which are associated with T helper type 2 (Th2) cells and are also required for restraining the production of systemic type-1 cytokine, can prevent lethal immunopathology during acute infection with *T. gondii* (Christopher et al., 2012). Therefore, the significant up-regulated of IFN-γ, IL-2, IL-12p40, IL-12p70, in combination with the slight increased production of IL-4 and IL-10 in all the immunized groups suggest that an appropriate Th1-type and Th2-type cellular response conferred host resistance to *T. gondii* infection. Furthermore, the highest levels of these cytokines induced by the two-gene cocktail DNA immunization in mice indicate that multi-gene DNA vaccine candidate could significantly enhance Th1-type immune responses that areinduced by a single-gene DNA vaccine.

CD4^+^ T and CD8^+^ T cells are activated once the intracellular parasite *T. gondii* invades the host cell and play critical roles in adaptive immune responses against *T. gondii* infection (Erik et al., 2010; Zhang et al., 2015). Especially, in synergy with CD4^+^ T lymphocytes, CD8^+^ T lymphocytes are key to limit *T. gondii* invasion during acute spreading of *T. gondii* tachyzoites or to limit the formation of brain cysts in the late stage of infection (Gigley et al., 2009; Christopher et al., 2012). In accordance with previous studies on DNA vaccination (Zhang et al., 2018), our results showed that the increased percentage of CD4^+^ and CD8^+^ T cells was observed in immunized groups, especially in two-gene immunized groups, suggesting that the DNA immunization with pVAX-MIC5 and or pVAX-MIC16 induces T lymphocytes, which contribute to the inhibition of the formation of tissue cysts in brain and restriction of *T. gondii* dissemination inside the body.

*T. gondii* strains belong to different genotypes with variable virulence in animals and humans (Sibley and Ajioka, 2008; Gigley et al., 2009). In this study, we used Kunming mice, which are highly susceptible to *T. gondii*, to evaluate the protective efficacy of DNA vaccine including pVAX-MIC5, and/or pVAX-MIC16 against *T. gondii* virulent RH strain (Type I) and avirulent PRU strain (Type II) challenge. As expected, TgMIC5 or TgMIC16 induced cross-protection against infection with different genotypes of *T. gondii*, which is similar to previous studies involving TgMIC13 and TgMIC6 (Yuan et al., 2013), indicating that these two TgMICs are good DNA vaccine candidates against *T. gondii* infection. However, the high levels of Th1-type based immune response and antibody response led to only partial protection against *T. gondii*, which may be ascribed to limited immunity to *T. gondii* induced by DNA vaccine.

In conclusion, the present study demonstrates that the recombinant plasmids encoding TgMIC5 and/or TgMIC16, are good DNA vaccine candidates, which induce immune protection against acute and chronic *T. gondii* infection in mice. Further studies are needed to investigate the protective immunity induced by using a combination with more *T. gondii* antigens.

## Data Availability Statement

The original contributions presented in the study are included in the article/supplementary material. Further inquiries can be directed to the corresponding authors.

## Ethics Statement

The animal study was reviewed and approved by the Animal Ethics Committee of the ethical committee of Ningbo University (permission: SYXK(ZHE)2013-0190).

## Author Contributions

Y-CZ and JC conceived and designed the study. Y-CZ, L-JM and JC performed the experiments and assisted in the preparation of the manuscript. Y-CZ, J-LZ, J-FL and YH analyzed the data. JC prepared the manuscript. Y-CZ and JC assisted in the design of the experiments, data interpretation, and manuscript preparation. All authors contributed to the article and approved the submitted version.

## Conflict of Interest

The authors declare that the research was conducted in the absence of any commercial or financial relationships that could be construed as a potential conflict of interest.

## Publisher’s Note

All claims expressed in this article are solely those of the authors and do not necessarily represent those of their affiliated organizations, or those of the publisher, the editors and the reviewers. Any product that may be evaluated in this article, or claim that may be made by its manufacturer, is not guaranteed or endorsed by the publisher.
